# Dynamic regulation of proximal tubular autophagy from injury to repair after ischemic kidney damage

**DOI:** 10.1186/s11658-024-00663-w

**Published:** 2024-12-05

**Authors:** Yuhong Gong, Wei Zhu, Yongqiang Li, Tao Lu, Jiexing Tan, Changsheng He, Luodan Yang, Yufeng Zhu, Li Gong

**Affiliations:** 1 Experimental Animal Center, Nanfang Hospital, Southern Medical University, Guangzhou, 510515 China; 2 Department of Infectious Diseases, State Key Laboratory of Organ Failure Research, Guangdong Provincial Key Laboratory of Viral Hepatitis Research, Nanfang Hospital, Southern Medical University, Guangzhou, 510515 China; 3 Department of General Practice, The Third Affiliated Hospital of Southern Medical University, Guangzhou, 510630 China; 4 Changzhou Geriatric Hospital Affiliated to Soochow University, Changzhou No. 7 People’s Hospital, Changzhou, 213011 China; 5 School of Physical Education and Sports Science, South China Normal University, Guangzhou, 510006 China

**Keywords:** Autophagy, Proximal tubule, Acute kidney injury, Interstitial fibrosis

## Abstract

**Background:**

The role of proximal tubular autophagy in repairing kidney injury following ischemia remains unclear.

**Methods:**

In this study, we utilized mice with conditional deletion of the *Atg5* gene in proximal tubules and monitored the long-term dynamic regulation of autophagy following ischemic acute kidney injury (AKI).

**Results:**

The results showed that *Atg5*-deficient proximal tubule epithelial cells exhibited damaged mitochondria, concentric membranes, and lysosomal accumulation 24 h after ischemia/reperfusion. However, 28 days after ischemia/reperfusion, concentric membrane bodies remained, but lysosomal accumulation was no longer observed. Notably, the absence of *Atg5* in renal tubular epithelial cells impaired renal function and led to increased tubular cell proliferation and oxidative stress in the early stage of injury. However, during the repair period following AKI, *Atg5* deficiency exhibited no significant difference in the expression of proliferating cell nuclear antigen (PCNA) and 4-hydoxynonenal (4HNE), suggesting that the improvement in renal fibrosis associated with *Atg5* deficiency is unlikely to result from its effect on cell proliferation or reactive oxygen species levels. Additionally, *Atg5* deficiency inhibits the secretion of profibrotic factor fibroblast growth factor 2 (FGF2) from the early stage of renal injury to the recovery stage of AKI, indicating that autophagy-specific regulation of FGF2 secretion is a dynamic process overlapping with other stages of injury. Furthermore, increased co-localization of ATG5 with 4HNE and FGF2 was observed in patient samples.

**Conclusion:**

In summary, our results suggest that the dynamic regulation of autophagy on key molecules involved in kidney injury and repair varies with the stage of kidney injury.

**Graphical Abstract:**

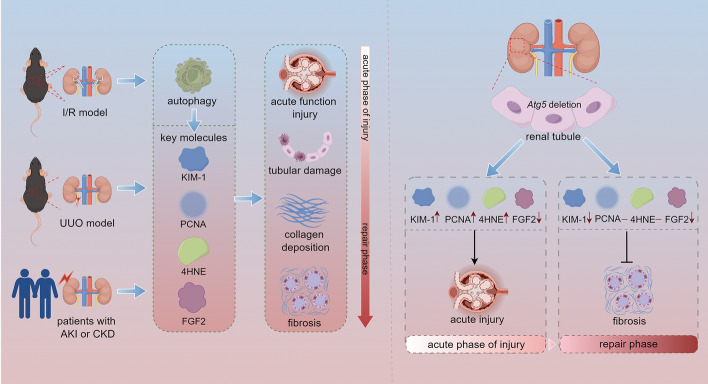

**Supplementary Information:**

The online version contains supplementary material available at 10.1186/s11658-024-00663-w.

## Introduction

Acute kidney injury (AKI) remains a critical global health issue, and patients who survive dialysis-requiring AKI have a significantly increased risk of developing chronic kidney disease (CKD), which can progress to end-stage renal disease (ESRD) [1–3]. Due to the complexity of the various molecules and pathways involved, the precise mechanisms driving AKI-CKD are poorly understood, and there is a lack of effective treatment options to prevent the occurrence and progression of acute kidney injury [4–7]. The precise mechanisms driving the AKI-CKD transition remain unclear. Therefore, understanding the potential mechanisms of AKI to CKD is crucial for developing effective CKD treatment interventions.

The pathogenesis of AKI involves various factors, including oxidative stress, hypoxia, inflammation, and tubule injury. These factors can result from toxins, drugs, and ischemia. In response to these pressures, autophagy acts as a cytoprotective mechanism by degrading and recovering damaged organelles and cytoplasmic components, thereby preventing the accumulation of damaged cellular components and reducing cell damage [8, 9]. Tubular cell injury, particularly tubular atrophy leading to renal fibrosis, is a primary cause and common pathological process of end-stage renal disease.

Studies over the past decade have elucidated the role of autophagy in tubular injury and repair following acute kidney injury. Under physiological conditions, autophagy is essential for maintaining cellular homeostasis in the proximal tubules. Additionally, autophagy is remarkably activated in tubular epithelial cells in response to various stressors in AKI models [10–14].

In the acute phase of injury, impairing autophagy by *Atg5* or *Atg7* deletion in the proximal tubules of mice worsened kidney function and increased tubular cell apoptosis, oxidative stress, and inflammatory responses compared to those in wild-type (WT) mice, suggesting a possible protective role of autophagy early in injury [15]. However, it remains unclear whether autophagy prevents or contributes to insufficient repair during the repair phase. Some studies have revealed that tubular autophagy increases profibrotic factors, promotes excessive collagen production, and exacerbates interstitial fibrosis [16–18]. In contrast, other studies have demonstrated that autophagy serves as an anti-fibrotic mechanism, reducing collagen production, and alleviating interstitial fibrosis [19, 20]. These conflicting findings suggest that the regulation of autophagy may vary depending on the phase or severity of injury. Moreover, most studies assessing autophagy have only examined one or two timepoints, limiting our understanding of the dynamic process of autophagy regulation in the progression from AKI to CKD.

In this study, we employed a proximal tubule-specific deletion of *Atg5* in mice to advance our understanding of the long-term dynamics of autophagy regulation following ischemic AKI. We found that *Atg5* deficiency increased tubular cell proliferation and reactive oxygen species (ROS) production early in injury. However, there was no difference between *Atg5* deficiency and WT mice during repair. Furthermore, we demonstrated that *Atg5* deficiency inhibits the secretion of the profibrotic factor fibroblast growth factor 2 (FGF2) from the early stages of kidney injury and that *Atg5* deficiency protects against the progression of AKI to CKD.

## Methods

### Animals

*Atg5*^flox/flox^ mice were purchased from the RIKEN BioResource Center (Tsukuba, Ibaraki, Japan). Phosphoenolpyruvate carboxy kinase-cAMP-response element (PEPCK-Cre) mice originally came from Dr. Haase at Vanderbilt University. Floxed *Atg5* from the C57BL/6 background was crossed with PEPCK-Cre mice to generate PEPCK-Cre^+^*Atg*5^flox/flox^ (PT-*atg5* KO) and PEPCK-Cre^−^*Atg5*^flox/flox^ (WT) mice. Tail samples were collected from mice at 4 weeks of age for genotyping using polymerase chain reaction (PCR). Primer sequences are listed in Table 1. Flox *Atg5* genotyping produced fragments of 350 bp for the WT and 700 bp for the mutant allele. WT mice showed only the 350 bp band, homozygous (*Atg5*^flox/flox^) displayed a 700 bp band, and heterozygous (*Atg5*^flox/+^) showed both bands. Cre-positive (Cre^+^) mice yields a 370 bp band, while Cre-negative (Cre^−^) mice showed no band. The mice were housed under specific pathogen-free (SPF) environment with ad libitum access to food and water.

**Table 1:** Primers for quantitative real-time PCR and genotyping

Gene name	Sequences
*Atg5* flox	Exon 3–1: 5′-GAATATGAAGGCACACCCCTGAAATG-3′
	Check 2: 5′-ACAACGTCGAGCACAGCTGCGCAAGG-3′
	Short 2: 5′-GTACTGCATAATGGTTTAACTCTTGC-3′
PEPCK Cre	Forward: 5′-ACCTGAAGATGTTCGCGATTATCT-3′
	Reverse: 5′-ACCGTCAGTACGTGAGATATCTT-3′
*Collagen I*	Forward: 5′-ACATGTTCAGCTTTGTGGACC-3′
	Reverse: 5′-TAGGCCATTGTGTATGCAGC-3′
*Gapdh*	Forward: 5′-GCCATCACTGCCACCCAGAA-3′
	Reverse: 5′-GCCAGTGAGCTTCCCGTTGA-3′

### Mouse model

Littermates of 8- to 10-week-old male mice were randomly assigned to either sham operation or model group. Before surgery, the mice were anesthetized via intraperitoneal injection of pentobarbital sodium (60 mg/kg). Anesthetized mice were placed on a thermostatic heating pad to maintain body temperature. The renal pedicles were separated and clamped for 25 min to establish a bilateral ischemia/reperfusion (I/R) AKI model (*n* = 5–10 per group). Mice were sacrificed at 24 h and 2, 4, 7, 14 and 28 days postreperfusion to collect kidney samples. In the unilateral ureteral obstruction (UUO) model, the ureter of the left kidney was exposed and ligated (*n* = 6 per group). After 14 days, the mice were sacrificed, and the left blocked kidneys were collected for further study.

### Analysis of serum creatinine and urea

Serum samples were collected at 24 h, and 2, 4, 7, 14, and 28 days post-AKI model establishment and analyzed using an automatic biochemical analyzer (Mindray, BS-240VET).

### ELISA measurement of urinary KIM-1 levels

Urine samples were collected at the indicated time and subsequently analyzed using the Mouse KIM-1 Elisa Kit (BOSTER, EK0880) according to the manufacturer’s instructions. Urinary KIM-1 was expressed as the ratio of KIM-1 concentration to creatinine concentration (ng/mg).

### Renal histology

The collected kidneys were fixed with 4% paraformaldehyde, embedded in paraffin, and sectioned to 4 μm thickness. Hematoxylin and eosin (H&E) staining was performed following the manufacturer’s instructions (LEAGENE, DH0020). Collagen deposition was evaluated using Masson’s trichrome staining kit (LEAGENE, DC0032) and Sirius Red staining kit (LEAGENE, DC0040) according to the manufacturer’s instructions. Images were captured using a PRECIC 500 B digital scanner. Six fields were randomly selected for each section for the quantitative analysis of collagen deposition using ImageJ software.

### Ultrastructural analysis

For ultrastructural analysis, the kidneys were sectioned into 1 mm^3^ blocks and sequentially fixed in 2.5% glutaraldehyde and 1% osmic acid. Following dehydration, the tissue was embedded in resin, and ultrathin sections were prepared and analyzed using a Hitachi H-7500 transmission electron microscope.

### Immunofluorescence

After dewaxing and hydration, sections were incubated with 3% hydrogen peroxide for 10 min at 25 ℃. Antigen retrieval was performed using citrate (pH 6.0) or Tris–EDTA (pH 9.0) followed by blocking with 3% BSA for 30 min. Primary antibodies were incubated at 37 °C for 2 h, then washed with PBS, followed by incubation with secondary antibodies for 30 min at 25 ℃. Samples were then incubated with iFluor™ 610 Styramide (1:150; AAT Bioquest, 44904) for 10 min in the dark. Lotus Tetragonolobus Lectin (LTL)-488 dye (1:150; Vector Laboratories, FL-1321-2) was incubated for 30 min and subsequently washed with PBS. Sections were counterstained with DAPI solution (1:150; Solarbio, C0065) for 10 min. The results were visualized using a microscope (ZEISS, LSM980) after coverslipes were applied. Six visual fields were randomly selected for each section, and ImageJ software was used to quantify positive areas. Antibodies against KIM-1 (1:50; R&D systems, AF1817), PCNA (1:50; Abcam, ab92552), α-SMA (1:50; Abcam, ab5694), VIMENTIN (1:200;Proteintech, 10366-1-AP), PDGFR-β(1:50; Thermofisher, A700-081), 4HNE (1:40; Arigobio, ARG23967), FGF2 (1:800; Abcam, ab72316), CTGF (1:50; Proteintech, 23936-1-AP), TGFβ1(1:500; Abcam, ab215715), goat anti-rabbit IgG-horseradish peroxidase (HRP; ZSGB-BIO, PV-6001), and goat anti-rat IgG-horseradish peroxidase (HRP; ZSGB-BIO, PV-9004) were used.

### Immunohistochemistry (IHC) of mouse kidneys

For immunohistochemical detection of fibronectin-1 (FN1), mouse kidneys were collected 28 days after bilateral I/R. Kidney tissue sections (4 μm) were incubated with 3% hydrogen peroxide to inactivate endogenous peroxidase, followed by antigen retrieval using citrate (pH 6.0) and blocking with 3% BSA. Rabbit anti-FN1 (1:200; Abcam, ab2413) was incubated at 4 °C overnight, followed by incubation with goat anti-rabbit IgG-horseradish peroxidase (HRP; ZSGB-BIO, PV-6001) secondary antibody for 40 min at 25 ℃. After washes, the signals were developed using a DAB kit and counterstained with hematoxylin. The images were acquired using a PRECIC 500 B digital scanner. Six fields were randomly selected per section for quantitative analysis of the FN1-positive area using the ImageJ software.

### Western blotting

Mouse kidney tissues were lysed with radioimmunoprecipitation assay (RIPA) lysis buffer (CWBIO, CW23335) containing protease inhibitors to obtain total protein. The extracted protein samples were separated by sodium dodecyl sulfate–polyacrylamide gel electrophoresis and transferred onto polyvinylidene fluoride (PVDF) membrane. The PVDF membranes were blocked with 5% skim milk in TBST buffer and subsequently incubated with primary antibodies overnight. After washing, the membranes were incubated with HRP-conjugated secondary antibody. Immunoblots were visualized with ECL primer western blotting detection reagents (Amersham, RPN2232) and photographed using a gel imaging system. Antibodies against β-ACTIN (1:1000; Sigma, A5361), ATG5 (1:1000; Cell Signaling Technology, 4691), p62 (1:1000; Cell Signaling Technology, 4060), LC3B (1:1000; Cell Signaling Technology, 9102), goat anti-rabbit IgG–horseradish peroxidase (HRP; 1:10,000), and goat anti-mouse IgG-horseradish peroxidase (HRP; 1:10,000) were used.

### Quantitative real-time PCR (qPCR)

Total RNA was extracted from kidney tissues and reverse-transcribed into complementary DNA for quantitative PCR (qPCR). Briefly, the cryopreserved kidney tissues were pulverized using a tissue grinder. TRIzol Reagent (Ambion, 15596026) was used to extract total RNA according to the manufacturer’s instructions. A commercial reverse transcription kit (Roche 4897030001) was used to generate cDNA. The reaction mixture (20 µL) of cDNA, primers, and SYBR Green (Roche, 04887352001) was subjected to qPCR using a Roche LightCycler 480 to evaluate *Collagen I* mRNA levels. The primer sequences used in this study are listed in Table 1.

### Morphological, 4HNE, FGF2 and autophagy detection of biopsy samples

Renal biopsy samples were obtained from six patients with minimal change disease (MCD), six patients with AKI, and six patients with chronic kidney disease (CKD) at Changzhou No. 7 People’s Hospital. AKI was defined according to the Kidney Disease: Improving Global Outcomes (KDIGO) guidelines [21]. Kidney biopsy samples were fixed, embedded, and sectioned at 3 μm sequentially. H&E, periodic acid-sliver methenamine (PASM) and Masson’s trichrome staining were performed for renal histology. We next detected the co-localization of ATG5, 4HNE and FGF2 in biopsy samples by immunofluorescence. The section incubated with 3% hydrogen peroxide and citrate (pH 6.0) for antigen repair, and blocked by incubation with 3% BSA, followed by exposure to rabbit anti-4HNE (1:40; Arigobio, ARG23967) or rabbit anti-FGF2 (1:800; Abcam, ab72316) at 37 ℃ for 2 h and Goat anti-Rabbit-HRP secondary antibody at 25 ℃ for 20 min. Then the samples were incubated with iFluor™ 610 Styramide (1:150; AAT Bioquest, 44904) for 10 min in the dark. After antigen retrieval in citrate again, the sections were incubated with rabbit anti-ATG5 (1:50; Abcam, ab108327) and secondary antibody subsequently. The signals were amplified by XFD546 tyramide reagent (1:150; AAT Bioquest, 11075). LTL and DAPI dyes were incubated for proximal renal tubules and nuclear visualization, respectively. The images were acquired using a microscope (ZEISS, LSM980). Six visual fields were randomly selected for each section, and ImageJ software was used for quantitative analysis of the positive areas.

### Statistical analysis

All experiments were conducted in a double-blind experiment. The data are expressed as mean ± SD. Student’s *t*-test was used for comparisons between two groups, and a one-way analysis of variance (ANOVA) test was used for multiple comparisons to determine significant differences among three or more groups. Statistical significance was set at *P* < 0.05. Statistical analysis was performed using GraphPad Prism 8.0.

## Results

### Establishment of selective ablation of *Atg5* in kidney proximal tubular mice.

**Fig. 1:**
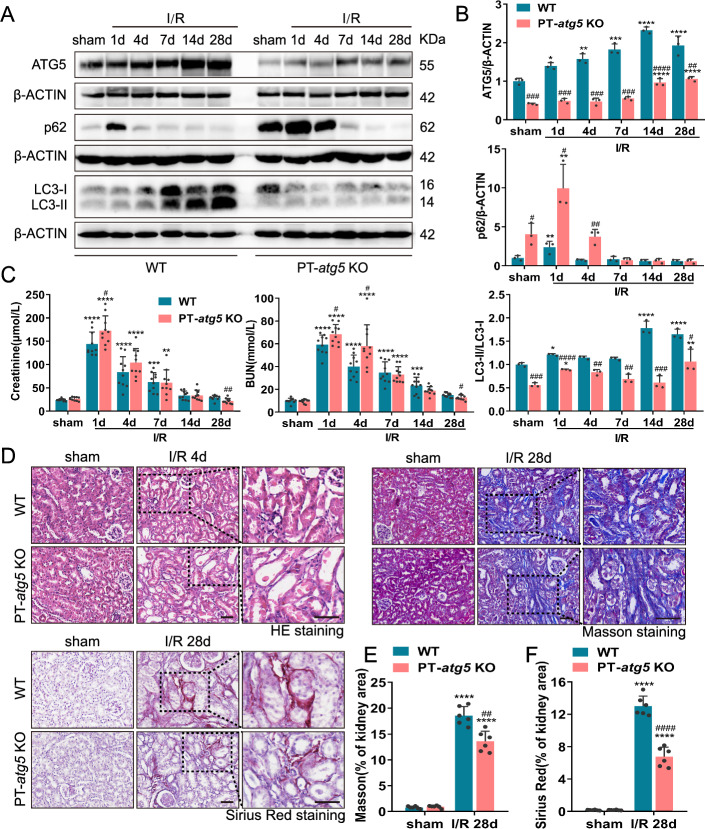
*Atg5* deletion in proximal tubules exacerbates acute renal injury but reduces fibrosis during the recovery phase after I/R. WT and PT-*atg5* KO mice were subjected to a sham operation or bilateral renal ischemia followed by reperfusion for up to 4 weeks (*n* = 5–10 mice per group), and the kidneys were collected at the indicated time points. **A**, **B** Representative immunoblots and quantification of ATG5, p62, and LC3 expression in the kidneys (*n* = 3). **C** Serum creatinine and BUN levels (*n* = 10). **D** Representative images of H&E staining, Masson’s trichrome and Sirius red staining. Scale bars, 50 μm. **E**, **F** Quantitative analysis of Masson’s trichrome and Sirius Red staining (*n* = 6). The values are expressed as mean ± standard deviation (SD), * represents a significant difference from the sham groups, and # represents a significant difference from the relevant wild-type group. # or * *P* < 0.05, ## or ** *P* < 0.01, ### or *** *P* < 0.001, #### or **** *P* < 0.0001

To further investigate the exact role of autophagy in the renal tubules, we established conditional deletion of *Atg5* to selectively ablate autophagy in proximal tubules. We crossed *Atg5*-floxed (*Atg5*^flox/flox^) mice with the PEPCK-Cre mice, establishing renal proximal tubule-specific *atg5* knockout (PT-*atg5* KO) mice. WT littermates were used as controls. The breeding protocol is illustrated in Supplementary Figure S1A. PCR genotyping was performed for each mouse, with the PT-*atg5* KO genotype confirmed by (1) amplification of the 700-bp fragment of the floxed allele, (2) lack of amplification of the 350-bp fragment of the WT allele, and (3) amplification of the 370-bp fragment of the Cre gene (Supplementary Figure S1B). Western blot analysis revealed significantly reduced ATG5 expression in PT-*atg5* KO mice compared to WT littermates, as well as markedly decreased LC3-I to LC3-II conversion in the kidney cortex and outer medulla (Fig. 1A, B). Furthermore, western blot analysis demonstrated the accumulation of p62, a selective substrate of autophagy, which was markedly higher in PT-*atg5* KO kidney tissues than in WT (Fig. 1A, B). These findings confirm the specific deletion of *Atg5* in renal proximal tubular epithelial cells in the PT-*atg5* KO model. Without surgical treatment, these mice exhibited comparable blood urea nitrogen (BUN) and serum creatinine levels to WT (Fig. 1C), indicating preserved renal function.

### *Atg5* deficiency in proximal tubules aggravates renal injury in the acute phase and attenuates renal fibrosis in the recovery period after AKI

AKI plays an important role in the onset and progression of CKD. The I/R model has been employed to investigate the mechanisms underlying the transformation from AKI to CKD. To analyze the impact of autophagy in the proximal tubules after ischemia-induced AKI, we first examined autophagy at the specified time points. As shown in Fig. 1A, B, WT kidney tissues showed continuous activation of tubular autophagy, whereas PT-*atg5* KO kidney tissues showed significantly reduced ATG5 expression, a decreased LC3II/I ratio, and an accumulation of p62 compared with WT littermate mice, suggesting that the activation of tubular autophagy was nearly abolished in PT-*atg5* KO mice.

**Fig. 2:**
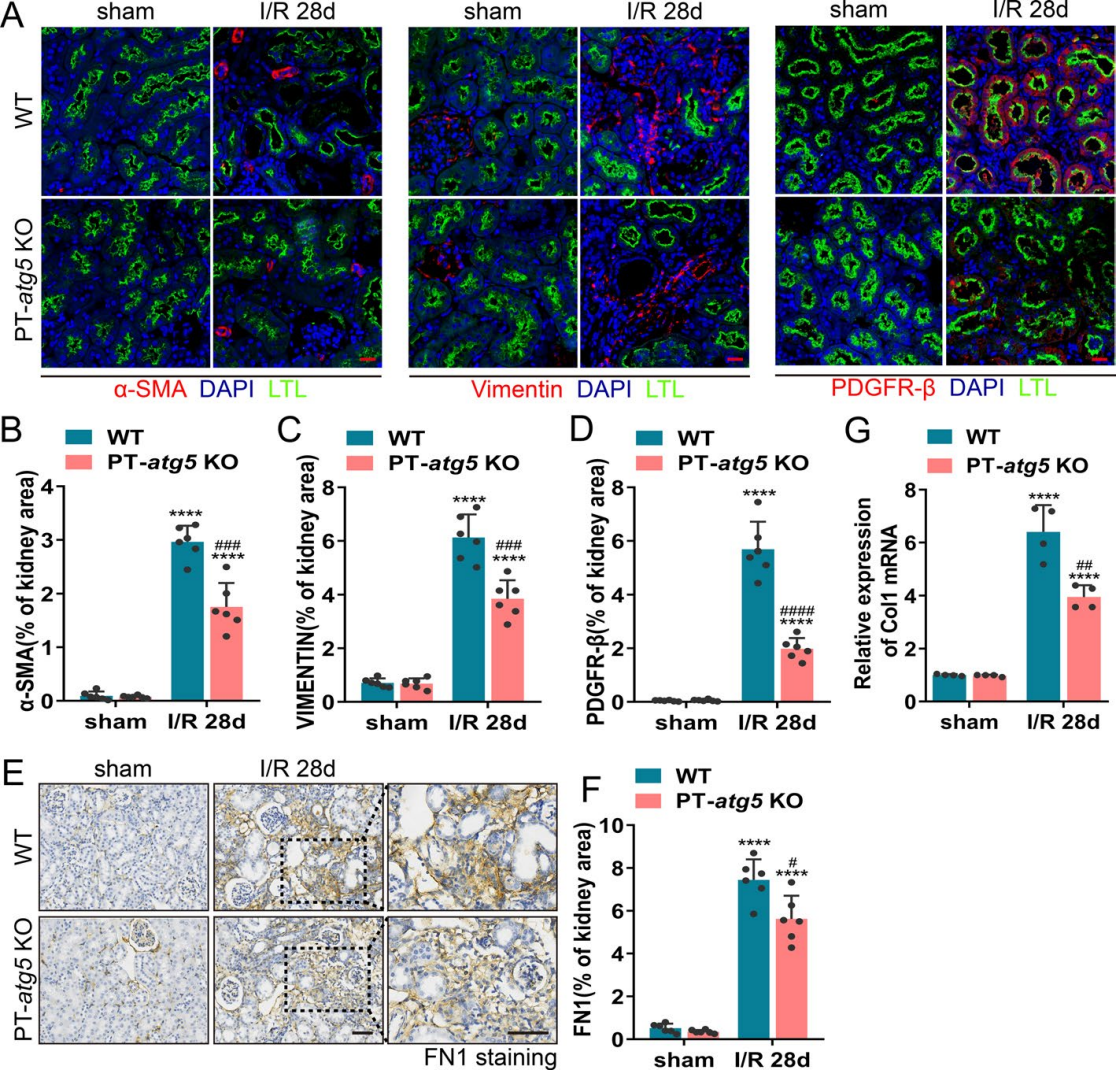
*Atg5* deficiency in proximal tubules suppresses renal interstitial fibrosis after I/R injury. Kidneys were collected from WT and PT- *atg5* KO mice after sham surgery or 4 weeks after bilateral renal ischemia/reperfusion. **A** Representative α-SMA (red, left), VIMENTIN (red, middle), and PDGFR-β (red, right) immunofluorescence staining images of kidney sections. The nuclei were stained with DAPI (blue). LTL was used to label proximal tubules (green). Scale bars, 20 μm. **B**–**D** Quantitative analysis of α-SMA, VIMENTIN, and PDGFR-β-positive area (*n* = 6). **E**, **F** The kidney sections were immunostained with antibody against FN1. The FN1-positive areas were quantified to view areas (*n* = 6). Scale bars, 50 μm. **G** mRNA levels of *collagen I* in kidney tissues, as detected by qPCR (*n* = 3). The values are expressed as mean ± SD. * represents a significant difference from the sham group; # represents a significant difference from the relevant wild-type group. # or * *P* < 0.05, ## or ** *P* < 0.01, ### or *** *P* < 0.001, #### or **** *P* < 0.0001

Subsequently, we examined the effects of autophagy deficiency on kidney function and morphology during ischemic AKI in PT-*atg5* KO mice. At 24 h post-renal ischemia/reperfusion, the deletion of *Atg5* in proximal tubular significantly worsened renal function compared to WT littermates, exhibiting severe brush edge loss and tubular dilation, which resulted in BUN and serum creatinine levels of 68.38 mmol/L and 172.98 μmol/L, respectively (Fig. 1C). In contrast, WT littermate mice demonstrated BUN levels of 59.26 mmol/L and serum creatinine levels of 144.24 μmol/L. At 48 h after ischemia/reperfusion, serum creatinine and BUN levels in both groups increased, but no significant differences were observed compared to the levels at 24 h (Supplementary Figure S2A). At day 4 post renal ischemia/reperfusion, PT-*atg5* KO mice had 58.12 mmol/L BUN and 104.50 μmol/L serum creatinine, while WT littermate mice had 39.98 mmol/L BUN and 83.71 μmol/L serum creatinine (significantly higher than the levels of the WT). Furthermore, renal histological examination revealed more severe tubule injury in PT-*atg5* KO mice compared with WT littermates (Fig. 1D, Supplementary Figure S2B). At 7 days or 2 weeks after AKI, serum creatinine and BUN levels in PT-*atg5* KO were closely comparable to those in WT mice. However, at 4 weeks after AKI, WT mice still exhibited 14.99 mmol/L BUN and 28.96 μmol/L serum creatinine level, whereas PT-*atg5* KO mice had 12.90 mmol/L BUN and 21.93 μmol/L serum creatinine level, indicating better renal recovery (Fig. 1C). Consistently, Masson’s trichrome and Sirius red staining revealed decreased collagen deposition in PT-*atg5* KO (Fig. 1D–F). PT-*atg5* KO mice also had less α-SMA (Fig. 2A, B), VIMENTIN (Fig. 2A, C), PDGFR-β (Fig. 2A, D), FN1 (Fig. 2E, F), and *collagen* *I* (Fig. 2G) than control group at day 28 after I/R. Collectively, the results indicate that *Atg5* deletion in proximal tubule aggravates renal injury during the acute phase while attenuating renal fibrosis during the recovery period following AKI.

### *Atg5* deletion in proximal tubules changes the ultrastructure of proximal tubules of the ischemic kidney

**Fig. 3:**
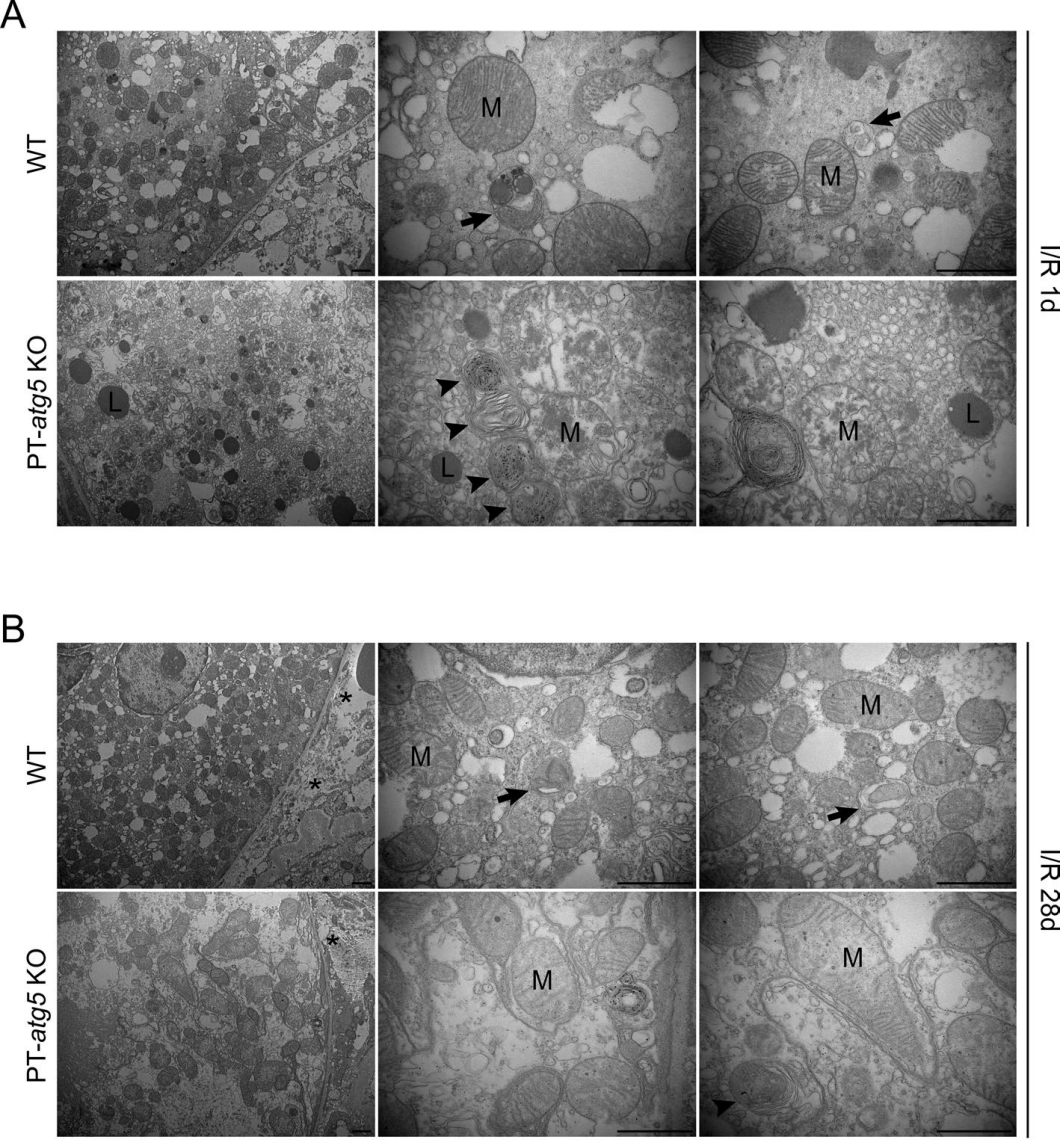
Proximal tubule *Atg5* deletion changes the ultrastructure of the proximal tubule in ischemic kidney injury. Electron micrographs of proximal tubule cells (**A**) 1 day after reflow and (**B**) 28 days after reflow following ischemia. At 1 day of I/R, the mitochondria from the PT-*atg5* KO group showed more severe cristae fragmentation and lysosomal storage than those from the WT group. Mitochondria-filled autophagosomes and autolysosomes were present in WT tubule cells, whereas concentric membranes were detected in *Atg5* deficient tubule cells. After 28 days, the mitochondria showed slight ridge fragmentation with intercellular collagen deposition. Autophagosomes and autolysosomes were still present in the WT group, and concentric membrane bodies were still detected in the PT-*atg5* KO group; however, lysosomal accumulation was no longer observed. The arrows indicate double-membrane autophagosomes and autolysosomes with a single membrane. Arrowheads indicate concentric membranes, asterisks indicate collagen fibers, mitochondria are labeled with “M,” and lysosomes are labeled with “L.” Scale bars, 1 μm

Kidneys were collected at 1 and 28 days post-ischemia/reperfusion for ultrastructural analysis to investigate the impact of *Atg5* on the tubule cell stress response. The results showed that proximal tubule epithelial cells exhibited variable sizes and deformed mitochondria 1 day after I/R. Mitochondria from the PT-*atg5* KO group showed more pronounced cristae fragmentation and effacement, along with lysosomal storage, compared with those from the WT group (Fig. 3A). Tubular epithelial cells of the WT group contained autophagosomes and autolysosomes filled with mitochondria, whereas concentric membrane bodies were detected around damaged mitochondria in *Atg5* deficient tubule cells (Fig. 3A). After 28 days, the mitochondria in both groups continued to exhibit mild cristae disruption, with collagen deposition observed between the cells (Fig. 3B). Autophagosomes and autolysosomes were still present in WT mice; in the PT-*atg5* KO group, concentric membrane bodies remained visible, but lysosomal accumulation was no longer observed.

### Characteristics of injury and repair in tubular autophagy-deficient mice after ischemic AKI

**Fig. 4:**
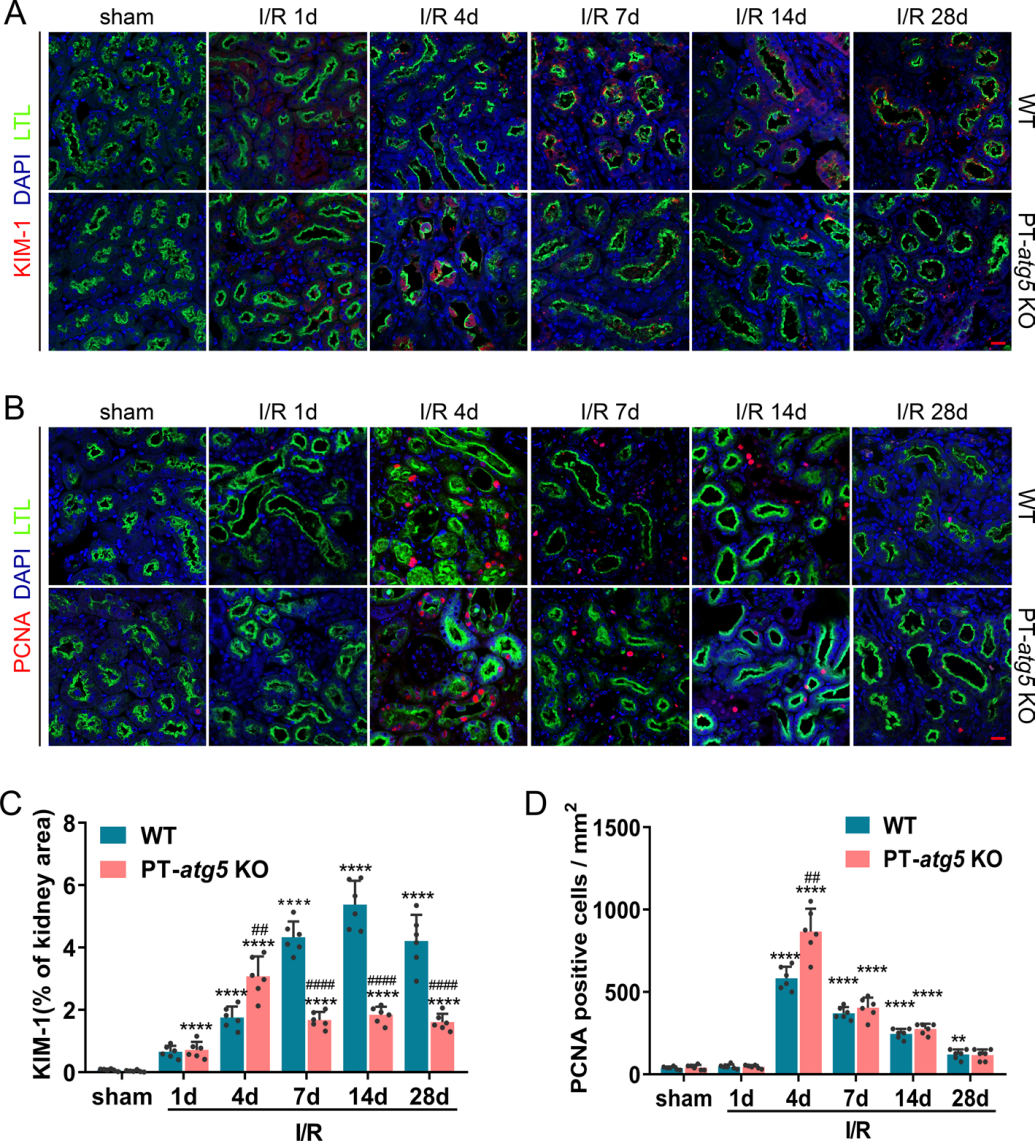
Dynamic changes of KIM-1 and PCNA in *Atg5*-deficient proximal renal tubules after I/R injury. WT and PT-*atg5* KO mice kidneys were harvested at 1, 4, 7, 14, and 28 days after sham surgery or bilateral ischemia/reperfusion, as described in Fig. 1, to observe the dynamic course of kidney injury and repair. **A** Immunofluorescence staining of KIM-1 (red) in the kidney sections. **B** Representative PCNA (red) immunofluorescence staining of kidney sections. Kidney tissue sections were stained with DAPI to visualize cell nuclei (blue) and LTL to visualize proximal tubules (green). Scale bars, 20 μm. **C** Quantitative analysis of the KIM-1 positive stained areas. **D** Quantification of PCNA-positive cells per mm^2^. The dates are shown as mean ± SD (*n* = 6). * Indicates significant difference from the sham group; # indicates a significant difference from the relevant wild-type group. # or *P < 0.05, ## or **P < 0.01, ### or ***P < 0.001, #### or ****P < 0.0001

To comprehensively investigate the effect of tubular *Atg5* deficiency on injury and repair mechanisms during AKI following I/R, we meticulously monitored the levels of kidney injury molecule-1 (KIM-1), a biomarker of renal tubular injury and proliferating cell nuclear antigen (PCNA), an indicator of cell proliferation. Immunofluorescent staining revealed that proximal tubule injury and cell proliferation notably increased in PT-*atg5* KO mice on day 4 following I/R injury compared to their WT counterparts (Fig. 4A, B), implying that the absence of tubular autophagy amplifies tubular damage and repair simultaneously during the acute injury stage. KIM-1 levels in WT kidneys continued to increase during the AKI repair process, peaking on day 14 (Fig. 4A, C), which may indicate a risk of progression from AKI to CKD [22–24]. In contrast, KIM-1 levels in PT-*atg5* KO mice remained stable on days 7, 14, and 28 after AKI, significantly lower than that in the WT group (Fig. 4A, C). Similar results were observed in urinary KIM-1 levels (Supplementary Figure S3A), further indicating that tubular *Atg5* worsened tubular damage during kidney recovery from AKI. Nevertheless, tubular *Atg5* seemed to be ineffective in cell proliferation during the repair stage, with no difference in the number of proliferating cells between the WT and KO groups at 7, 14, or 28 days after AKI. (Fig. 4B, D).

**Fig. 5:**
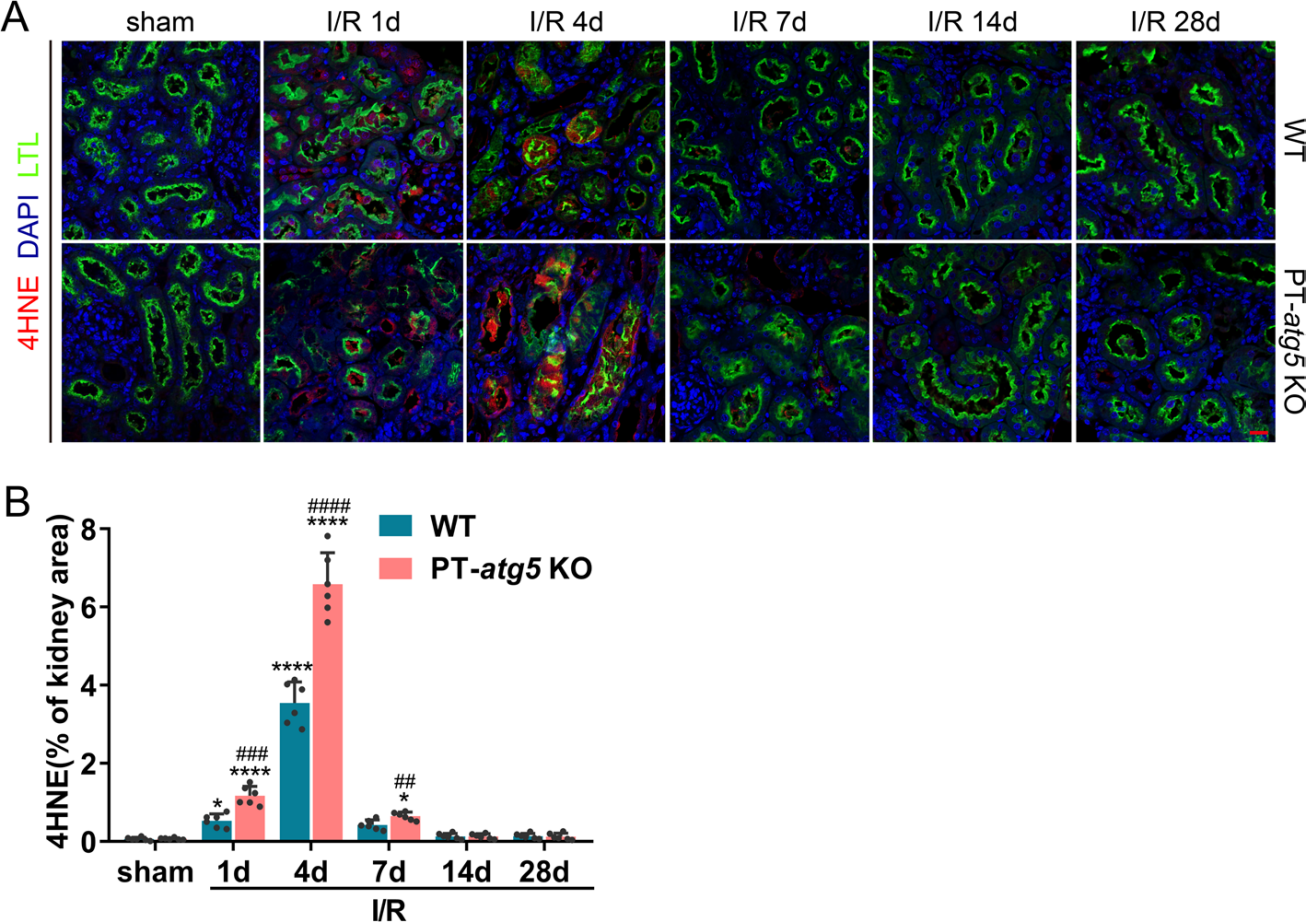
*Atg5* deficiency promotes 4HNE production in renal tubules in the acute injury phase of AKI. WT and PT-*atg5* KO mice were subjected to a sham operation or bilateral renal ischemia/reperfusion, and the kidneys were collected at the indicated time points for immunofluorescence analysis of 4HNE, a biomarker of oxidative stress. **A** Representative immunofluorescence staining images with the 4HNE antibody (red). The cell nuclei were labeled with DAPI (blue). Proximal renal tubules were stained with LTL (green). Scale bars, 20 μm. **B** Quantification of the 4HNE positive stained areas (*n* = 6). The values are expressed as mean ± SD, * Indicates a significant difference from the sham groups; # indicates a significant difference from the relevant wild-type group. # or *P < 0.05, ## or **P < 0.01, ### or ***P < 0.001, #### or ****P < 0.0001

Oxidative stress is significantly relevant to renal injuries, primarily during reperfusion [25]. To investigate the effect of renal tubular *Atg5* on oxidative stress during AKI progression, immunofluorescence was performed to detect the formation of 4-hydroxynonenal (4HNE) adduct, one of the most commonly used biomarkers for oxidative stress. A marked increase in tubular 4HNE adduct formation was observed on days 1 and 4 after I/R, further exacerbated by *Atg5* deletion (Fig. 5A, B). Fourteen and 28 days after I/R, there was no difference in the protein adduct formation of 4HNE between the WT and PT-*atg5* KO groups, with levels reduced those similar to the sham group (Fig. 5A, B), suggesting *Atg5* depletion in renal proximal tubules exacerbated tubular oxidative stress in the early phase of AKI following I/R, potentially contributing to the more severe kidney injury without autophagy.

### *Atg5* depletion in renal proximal tubules inhibits the production of tubular profibrotic factors after I/R

**Fig. 6:**
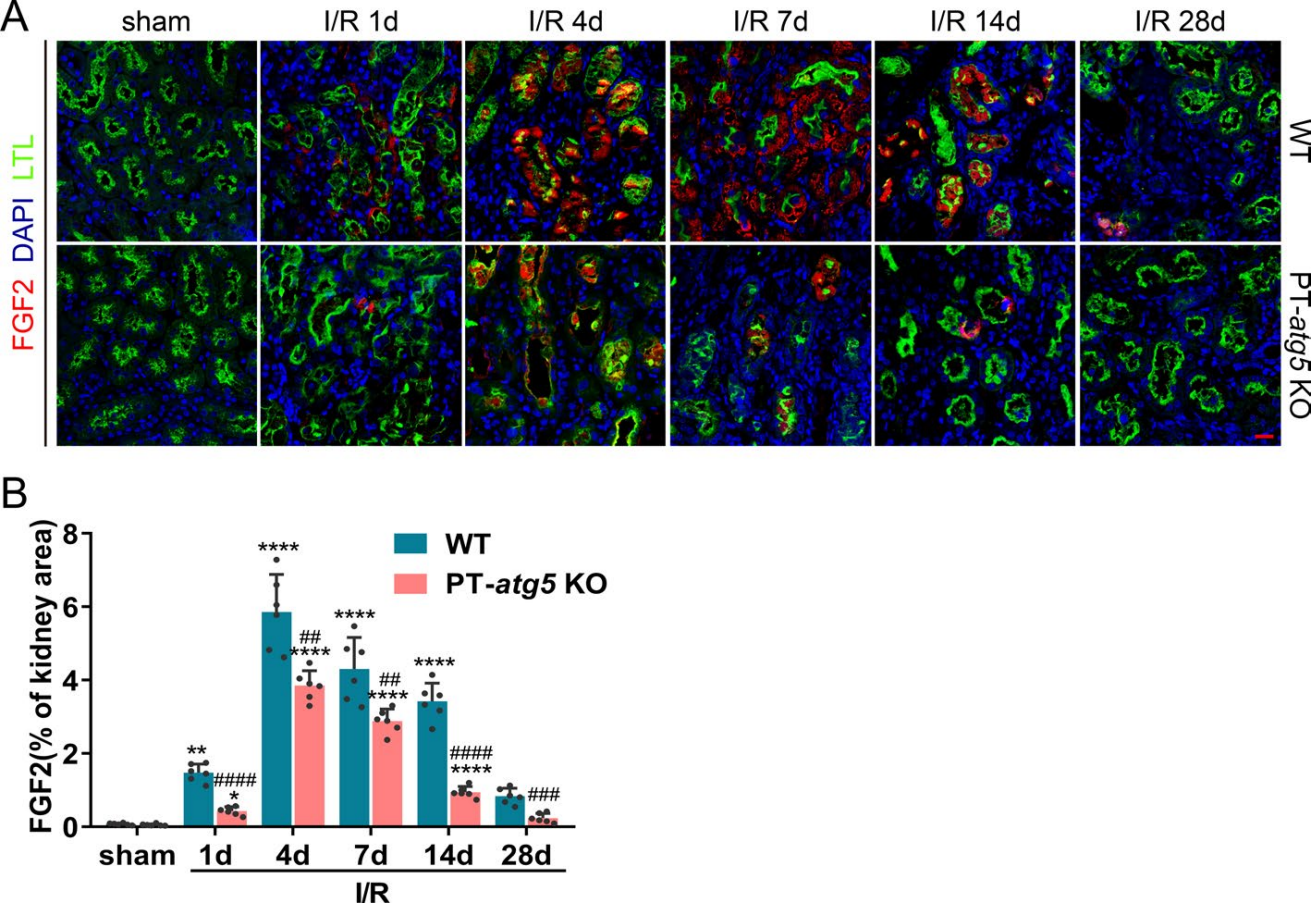
*Atg5* deletion inhibits FGF2 production in renal tubules after I/R. The kidneys of WT and PT-*atg5* KO mice were harvested at the indicated time points after sham surgery or bilateral I/R injury to detect the levels of the profibrotic factor FGF2. **A** Kidney slices were immunostained with antibody against FGF2 (red), DAPI to visualize the cell nuclei (blue), and LTL to visualize the proximal tubules (green). Scale bars, 20 μm. **B** Quantitative analysis of FGF2 positive areas. The dates are expressed as mean ± SD (*n* = 6). * represents a significant difference from the sham group; # represents a significant difference from the relevant wild-type group. # or *P < 0.05, ## or **P < 0.01, ### or ***P < 0.001, #### or ****P < 0.0001

Damaged tubular cells have the capacity to produce various growth factors, including FGF2, platelet-derived growth factor beta (PDGF-β), connective tissue growth factor (CTGF), and transforming growth factor beta 1(TGFβ1). These factors are well-documented for their role in promoting fibrosis formation during kidney recovery from AKI [18, 26–30]. The levels of theses factors were dynamically assessed during AKI progression. Tubular FGF2 levels in both groups increased rapidly after I/R, peaked on day 4, and then decreased and returned to baseline levels on day 28. Notably, during AKI progression, FGF2 production in the PT-*atg5* KO group was consistently significantly lower than that in WT kidneys (Fig. 6A, B), suggesting that *Atg5* deletion in tubule cells inhibited the production of tubular FGF2 after I/R. Additionally, the levels of PDGF-β (data not shown), TGFβ1, and CTGF also significantly increased after I/R and remained elevated throughout the AKI phase (Supplementary Figure S4A–D). However, there were no differences in PDGF-β (data not shown), TGFβ1 and CTGF levels between the WT and PT-*atg5* KO groups during the development of AKI (Supplementary Figure S4A–D), indicating that the absence of *Atg5* in renal tubules did not affect the production of renal PDGF-β, TGFβ1, and CTGF after I/R.

These results suggest that *Atg5* in renal proximal tubules is involved in regulating the production of profibrotic factors FGF2 after I/R, but not PDGF-β, TGFβ1, and CTGF.

### *Atg5* in renal proximal tubules regulates fibrosis in the UUO model

**Fig. 7:**
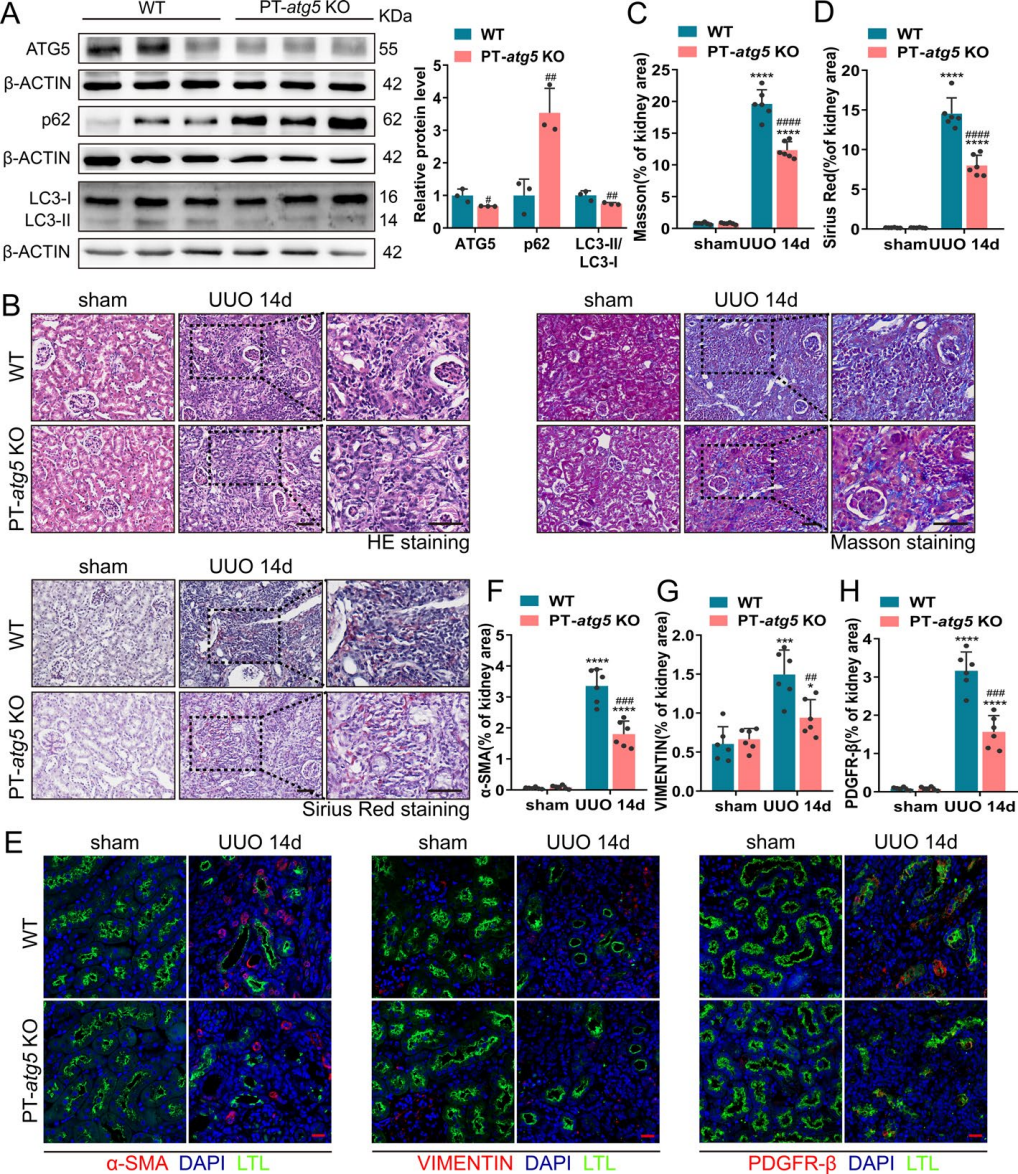
*Atg5* deficiency in renal proximal tubules inhibited interstitial fibrosis during UUO. WT and PT-*atg5* KO mice were subjected to sham operation or unilateral ureteral obstruction (UUO) (*n* = 6 each group), and the kidneys were harvested 14 days later. **A** Representative immunoblots and quantification of ATG5, p62, and LC3 expression in the kidneys (*n* = 3). **B** Representative images of H&E staining, Masson’s trichrome staining, and Sirius red staining. Scale bars, 50 μm. **C**, **D** Quantitative analysis of collagen deposition. **E** Kidney slices were immune stained with antibodies against α-SMA (red, left), VIMENTIN (red, middle), and PDGFR-β (red, right), labeled by DAPI to visualize cell nuclei (blue) and LTL to visualize proximal tubules (green). Scale bars, 20 μm. **F**–**H** Quantitative analysis of α-SMA, VIMENTIN, and PDGFR-β positive areas. The values are expressed as mean ± SD (*n* = 6). * Indicates a significant difference from the sham group; # indicates a significant difference from the relevant wild-type group. # or * *P* < 0.05, ## or **P < 0.01, ### or ***P < 0.001, #### or ****P < 0.0001

**Fig. 8:**
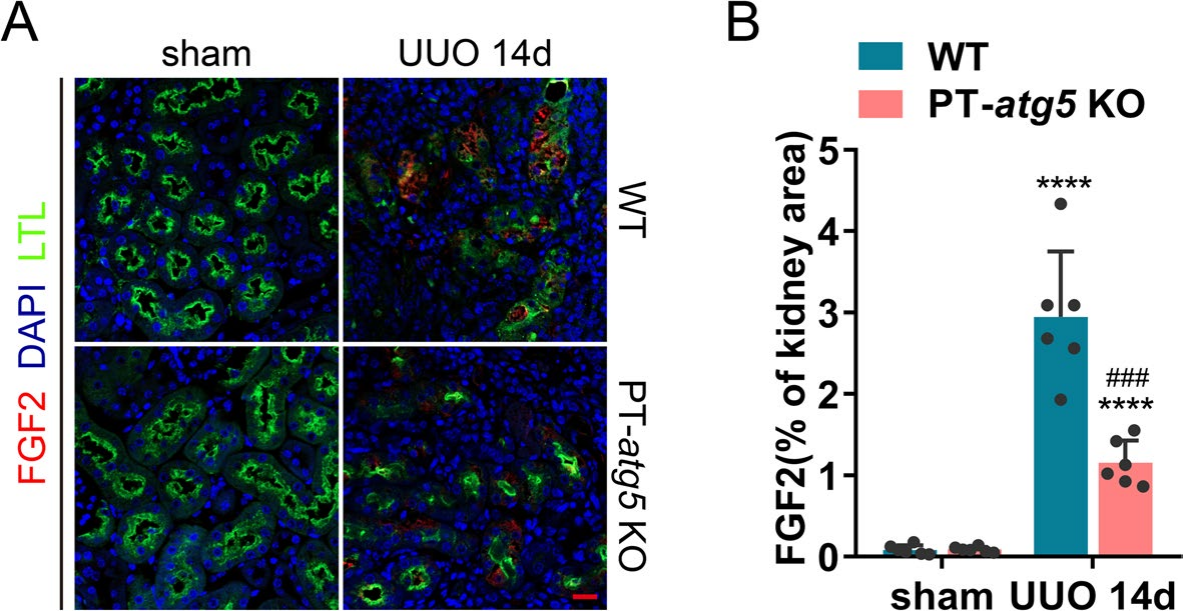
*Atg5* deficiency suppresses FGF2 production in renal tubules during UUO. Kidneys subjected to sham or UUO surgery were collected on day 14, as shown in Fig. 7. **A** Representative images of immunofluorescence staining images with FGF2 antibody (red). The cell nuclei were labeled with DAPI (blue). Proximal renal tubules were stained with LTL (green). Scale bars, 20 μm. **B** Quantification of FGF2 positive stained areas. The values are expressed as mean ± SD (*n* = 6). * Indicates a significant difference from the sham group; # indicates a significant difference from the relevant wild-type group. # or *P < 0.05, ## or ** *P* < 0.01, ### or ***P < 0.001, #### or ****P < 0.0001

To further verify the effect of *Atg5* in renal proximal tubules on renal fibrosis, we established a UUO model by ligating the left ureter, a classic model for studying renal interstitial fibrosis. On day 14 post-surgery, the expression levels of ATG5 and LC3B in the PT-*atg5* KO group were significantly lower than that in the WT group, while P62 accumulated in large amounts (Fig. 7A), indicating that successful elimination of the proximal tubule *Atg5* resulted in an impaired ability to induce autophagy in PT-*atg5* KO mice during UUO. The kidneys of the WT mice exhibited more severe structural disorders, characterized by tubular atrophy, intratubular cast formation, and inflammatory cell infiltration (Fig. 7B). Correspondingly, the KO group exhibited reduced collagen deposition (Fig. 7B–D) and lower levels of VIMENTIN, PDGFR-β, and α-SMA (Fig. 7E–H), primarily concentrated in the interstitial region, suggesting that tubule *Atg5* loss inhibited the formation of interstitial fibrosis during UUO. We also examined the induction of tubular profibrotic factors in the UUO model. *Atg5* deletion in proximal renal tubules inhibited the production of FGF2 (Fig. 8A, B), while PDGF-β (data not shown), CTGF (Supplementary Fig. 5A, C), and TGFβ1 (Supplementary Figure S5B, D) levels remained unaffected. These findings suggest that proximal tubule *Atg5* may exacerbate renal fibrosis in the UUO model by promoting the production of the profibrotic factor FGF2.

### Development of AKI and CKD from patients correlated with *Atg5*, oxidative stress, and profibrotic factors FGF2

**Fig. 9:**
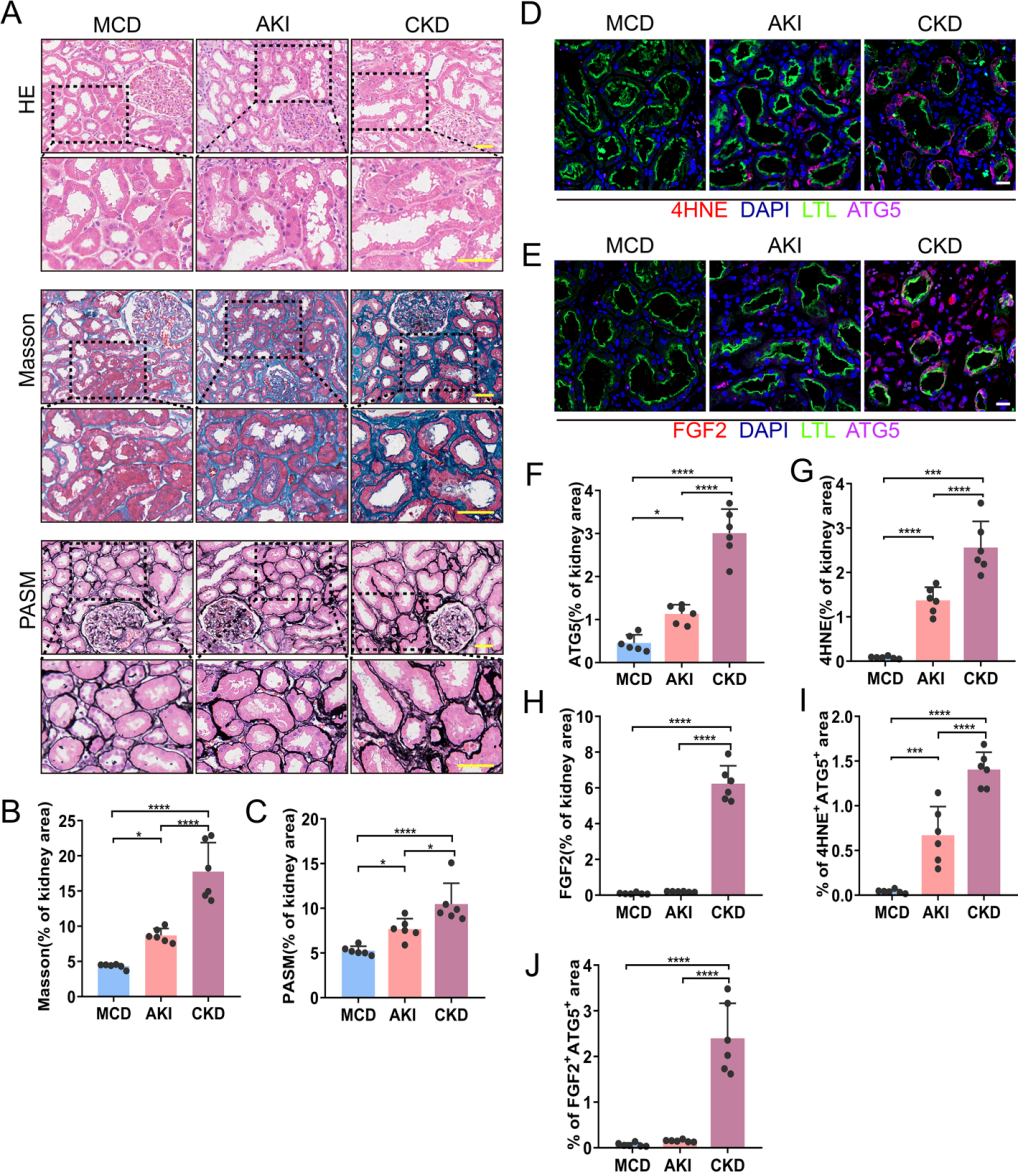
Increased induction of renal tubule autophagy, 4HNE, and FGF2 in renal biopsies in patients with AKI and CKD. Renal biopsies from patients with minimal change disease (MCD) as controls (*n* = 6), patients with AKI (*n* = 6), and patients with CKD (*n *= 6) were evaluated. **A** Representative images of H&E staining, Masson’s trichrome staining and PASM staining. Scale bars, 50 μm. **B**, **C** Quantitative analysis of Masson’s trichrome and PASM staining. **D** 4HNE (red) and ATG5 (violet) cells. **E** FGF2 (red) and ATG5 (violet) double immunofluorescence staining of the kidney sections. Cell nuclei were counterstained with DAPI (blue). Proximal renal tubules were stained with LTL (green). Scale bars, 20 μm. **F** Quantification of ATG5 positive area fraction. **G** Quantification of the 4HNE positive stained areas. **H** Quantification of FGF2 positive stained areas. **I** Quantification of 4HNE and ATG5 co-stained areas. **J** Quantification of FGF2 and ATG5 co-positive areas. The values are expressed as mean ± SD, *P < 0.05, **P < 0.01, ***P < 0.001, ****P < 0.0001

We sought to verify whether *Atg5* plays a similar role in regulating AKI and interstitial fibrosis in human samples. Our study included six patients with minimal change disease (MCD) as the control group, six cases of AKI, and six cases of CKD. Compared with MCD, both AKI and CKD exhibited more severe structural disorders, tubular damage, and increased collagen deposition, especially in CKD, as confirmed by Masson and PASM staining (Fig. 9A–C). Immunofluorescence results revealed persistent activation of ATG5 during AKI and CKD in human samples (Fig. 9D, F). Unlike the findings in the mouse model, the production of the oxidative stress marker 4HNE adduct increased not only during AKI but also during CKD (Fig. 9D, G), and the area co-stained by ATG5 and 4HNE increased significantly in AKI and CKD (Fig. 9D, I). The profibrotic factor FGF2 was found to be elevated exclusively in the renal tubules of patients with CKD (Fig. 9E, H). Similarly, the area co-stained with ATG5 and FGF2 significantly increased in the CKD group (Fig. 9E, J). Biopsy analyses of these patients showed that renal tubule autophagy, oxidative stress, and the profibrotic factor FGF2 were involved in the development of AKI and CKD.

## Discussion

Autophagy is a dynamic and continuous cellular process that serves as an adaptive response in renal tubule cells during AKI and renal interstitial fibrosis [31, 32]. The autophagic response in proximal tubular cells is not an isolated occurrence; rather, it involves multiple pathways and is part of continuous process [13, 33, 34]. However, most current research is limited to one or two timepoints, and these studies are inconsistent or controversial.

Autophagy is crucial for maintaining the structural and functional integrity of renal tubular epithelial cells under normal conditions [35, 36]. Autophagy is remarkably activated in proximal tubular epithelial cells in renal proximal tubules after ischemic damage [10, 12, 17, 37, 38]. In this study, we found that *Atg5* deletion in the proximal tubule aggravated renal function and injury during the acute phase of AKI while attenuating renal function and fibrosis during the repair period after AKI. These changes raise the question of whether they are associated with tubular injury and repair. Therefore, we studied the dynamic molecular events related to injury or fibrosis during the progression of AKI to CKD.

During the acute phase of AKI, we found that *Atg5* deficiency in the renal tubules maintained higher levels of proliferation accompanied by higher KIM-1 and ROS levels 4 days after I/R injury, suggesting that autophagy defects simultaneously increase tubular damage and regeneration in the early stages of I/R injury. Interestingly, *Atg5* deficiency cells increased PCNA expression early in injury, but there was no difference in PCNA expression between *Atg5* deficiency and WT during repair. Liu et al*.* found that the loss of *Atg5* significantly accelerates tubule cell apoptosis and proliferation on days 2 and 7 after I/R [10]. Li et al*.* also confirmed that a decrease in autophagy increased tubular cell proliferation 3 days after I/R injury [39]. We and other research groups have found that 4HNE were upregulated during the acute phase of AKI [40]. We further found that *Atg5* knockout in the renal proximal tubule increased 4HNE accumulation in the AKI injury phase, but there was no difference in 4HNE expression during the repair phase. In addition, Liu et al*.* found that the loss of autophagy in distal tubules of kidney promoted the increase of 4HNE aggregation but did not affect kidney function [10]. The above results suggest that the increase of 4HNE aggregation caused by *Atg5* deletion in proximal tubules may be one of the reasons for the aggravation of early AKI injury. In addition, ROS serves as a crucial signaling molecule in regulating cell proliferation. An increase in intracellular ROS can promote cell proliferation by activating various signaling pathways or through direct oxidative reactions with key proteins [41].

Several studies have reported that the absence of *Atg5* in the tubular epithelial cells leads to the formation of concentric membrane bodies and deformed mitochondria [10]. Using electron microscopy, we showed that the deletion of *Atg5* in tubular epithelial cells resulted in damaged mitochondria and concentric membranes and accumulation of lysosomes at 24 h after I/R, indicating that *Atg5* deficiency promotes lysosomal aggregation in the early stage of I/R. However, at 28 days after I/R, damaged mitochondria showed partial recovery, accumulation of lysosomes disappeared, and collagen fibers were reduced. Mitochondria are the main source of intracellular reactive oxygen species, and the sites of these reactive oxygen species production are very close to lysosomes. Xu et al. found that the lysosomal protein TRPML1 is a receptor for oxidative stress in cells. When activated, it can promote autophagy activity in cells, accelerate the clearance of damaged mitochondria, and reduce the production of free radicals by mitochondria [42].

Based on our data, we reasonably speculate that in the early stages of injury, mitochondria with autophagy defects release more reactive oxygen species, which promotes cell proliferation and leads to a large accumulation of activated lysosomes and accelerates the clearance of damaged mitochondria, and in turn reduces the release of reactive oxygen species. As suggested by Xu’s research, free radicals are a double-edged sword that can cause cell damage and activate protective mechanisms. This is precisely the mode of free radical feedback regulation [42]. This also explains well that the elevated 4HNE and PCNA in the autophagy-deficient group recovered to the level of the WT group in the later stage.

During kidney repair of AKI, whether autophagy is profibrotic or anti-fibrotic in maladaptive kidney repair has been controversial for many years [43–47]. Our results showed that *Atg5* is profibrotic in renal fibrosis induced by AKI and UUO. However, Li et al. demonstrated that *Atg5*-mediated autophagy in renal proximal epithelial cells inhibited renal fibrosis. We are not sure whether the different Cre tool mice cause the opposite result [47]. Baisantry et al. found that mice with specific *Atg5* knockout in the S3 tubular segment exhibited increased cell death 2 h after reperfusion but lower levels of inflammation, tubular senescence, interstitial fibrosis, and superior renal function 30 days after ischemic AKI, indicating that autophagy is a profibrotic factor in a mouse model of renal fibrosis after ischemia [16]. In our study, we observed *Atg5* deficiency can specifically inhibit the increase in FGF2 expression caused by I/R and UUO but has no significant effect on other profibrotic factors such as TGFβ1, PDGF-β, and CTCF. Livingston et al. also reported similar findings in *Atg7* deficient mice. To eliminate the interference of autophagy in early AKI injury, Livingston et al. established an inducible tubular-specific *Atg7* knockout mouse model, which only blocks tubular autophagy during renal repair, and the results still suggest the role of autophagy in promoting renal fibrosis [18]. Mechanistically, Livingston et al. further demonstrated that autophagy activates MAPK/ERK in renal tubular cells, thereby inducing EGR1 transactivation of FGF2 [48]. In our study, although *Atg5* deficiency in the proximal tubules exacerbates acute renal injury, the feedback regulation mechanism of ROS rapidly restores early injury to control levels without affecting the improvement of renal fibrosis during the repair phase. Notably, FGF2 expression increased from the early stages of ischemia/reperfusion injury in the I/R model, suggesting that FGF2 production is a dynamic process that overlaps with other stages. FGF2 is a regulatory factor for fibroblasts, and some studies have shown that FGF2 and FGFRs cell signaling is positively and negatively regulated by two different types of sphingolipids (Gb4, GM1), which promote fibroblast remodeling into different cell morphologies [49]. In this study, *Atg5* and *Atg7* belong to different ubiquitination-binding systems during vesiculation. Although they are both core autophagy-related proteins, further research is needed to determine whether their effects on FGF2&FGFR signaling are similar or not.

Clinically, we detected the induction of ATG5, 4HNE, and FGF2 in renal tubular epithelial cells from patients with AKI and CKD. Additionally, we observed increased co-localization of ATG5 with 4HNE and FGF2, indicating the potential mechanism of autophagy and related genes may contribute to the fibrosis development. Our research findings suggest that when treating renal fibrosis with gene editing, pharmacological agents, or small molecule compounds targeting autophagy and/or autophagy-related signaling, it is necessary to prevent excessive activation of ROS to eliminate its negative impact on treatment. In the future, further in-depth research is needed to explore the comprehensive network of autophagy and fibrosis-related pathways. This will enhance our understanding of the pathophysiological mechanisms underlying the transformation from AKI to CKD and provide a theoretical basis for the developing new treatment methods and intervention measures.

## Conclusion

This study highlights the critical role of autophagy in the progression from injury to repair following ischemic kidney damage. Autophagy dynamically regulates key molecules involved in kidney injury and repair at different stages of kidney injury. These findings suggest when treating renal fibrosis with targeting autophagy and/or autophagy-related signaling, it is necessary to prevent excessive activation of ROS to eliminate its negative impact on treatment.

## Supplementary Information

Supplementary Material 1: Figure S1. Establishment and identification of PT-*atg5* KO mice. (A) Breeding protocol for generating PT- *atg5* KO mice. Male littermate mice, 8–10 weeks old, were used for experiments after genotypes were confirmed. (B) Representative images of *Atg5* flox and PEPCK-Cre determination.

Supplementary Material 2: Figure S2. The effect of tubular *Atg5* deficiency on acute kidney injury after I/R. (A) Serum creatinine and BUN levels during acute injury phase (*n* = 5). (B) H&E staining images of the kidney at 2 days after I/R. Scale bar, 50 μm. The values are expressed as mean ± SD.

Supplementary Material 3: Figure S3. Tubule *Atg5* specific deletion affect the levels of urinary KIM-1 after I/R. Urine samples were collected at indicated times after sham surgery or after reperfusion following 25 min of bilateral ischemia. (A) Urinary KIM-1 normalized to urine creatinine (*n* = 3). The values are expressed as mean ± SD. # Represents a significant difference from the relevant wild-type group. # *P <*0.05.

Supplementary Material 4: Figure S4. *Atg5* deficiency does not affect the production of CTGF and TGFβ1 in renal tubules during renal repair after I/R. Kidneys with or without bilateral renal I/R were collected at indicated time points and processed for immunofluorescence analysis of profibrotic factor CTGF and TGFβ1. (A) Immunofluorescence staining for CTGF (red). (B) Kidney slices were immunostained with antibody against TGFβ1 (red), stained using DAPI to visualize cell nuclei (blue), and LTL to visualize proximal tubules (green). Scale bars, 20 μm. (C) Quantification of CTGF-positive area (%). (D) Quantitative analysis of TGFβ1 positive area fraction. The values are expressed as mean ± SD (*n* = 6). * represents a significant difference from the sham group; # represents a significant difference from the relevant wild-type group. # or * *P <*0.05, ## or ** *P <*0.01, ### or *** *P <*0.001, #### or **** *P <*0.0001.

Supplementary Material 5: Figure S5. Tubule *Atg5* specific deletion does not affect the production of CTGF and TGFβ1 in renal tubules during UUO. Kidneys subjected to sham or UUO surgery were collected on day 14 to measure levels of profibrotic factors TGFβ1 and CTGF. (A) Representative immunofluorescence staining images with the CTGF antibody (red). (B) Representative TGFβ1 (red) immunofluorescence staining images of kidney slices. The cell nuclei were labeled with DAPI (blue). Proximal renal tubules were stained with LTL (green). Scale bars, 20 μm. (C) Quantification of CTGF-positive areas. (D) Quantitative analysis of TGFβ1 positive area fraction. Scale bars, 20 μm. The values are expressed as mean ± SD (*n* = 6). * represents a significant difference from the sham group; # represents a significant difference from the relevant wild-type group. # or * *P <*0.05, ## or ** *P <*0.01, ### or *** *P <*0.001, #### or **** *P <*0.0001.

## Data Availability

The data that support the findings of this study are available on request from the corresponding author. Clinical data are not publicly available due to ethical restrictions approved by the ethics committee of Changzhou No. 7 People’s Hospital.

## Abbreviations

4HNE: 4-Hydoxynonenal
AKI: Acute kidney injury
BUN: Blood urea nitrogen
CKD: Chronic kidney disease
CTGF: Connective tissue growth factor
ESRD: End-stage renal disease
FGF2: Fibroblast growth factor 2
FN1: Fibronectin-1
H&E: Hematoxylin and eosin
I/R: Ischemia/reperfusion
KIM-1: Kidney injury molecule-1
LTL: Lotus tetragonolobus lectin
MCD: Minimal change disease
PASM: Periodic acid-sliver methenamine
PCNA: Proliferating cell nuclear antigen
PDGF-β: Platelet derived growth factor beta
PDGFR-β: Platelet derived growth factor receptor beta
PVDF: Polyvinylidene fluoride
ROS: Reactive oxygen species
TGFβ1: Transforming growth factor beta 1
UUO: Unilateral ureteral obstruction
WT: Wild-type
