# Supplementary figures and images for: Dynamic regulation of proximal tubular autophagy from injury to repair after ischemic kidney damage
Source: Cell Mol Biol Lett. 2024 Dec 5;29:151. doi: 10.1186/s11658-024-00663-w (PMC11619129; doi:10.1186/s11658-024-00663-w)

A

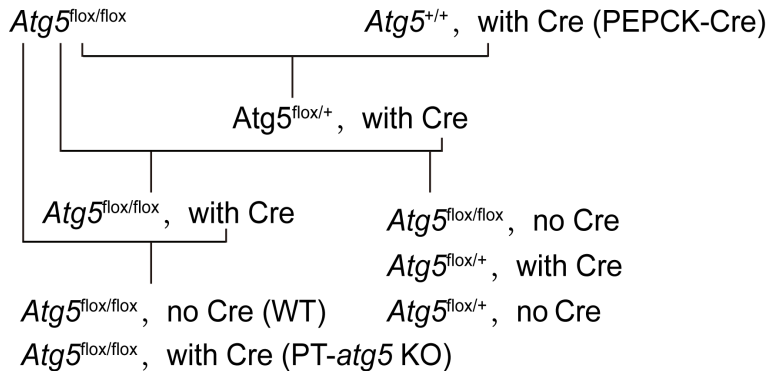

B

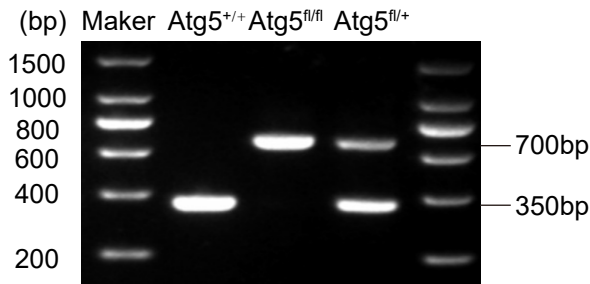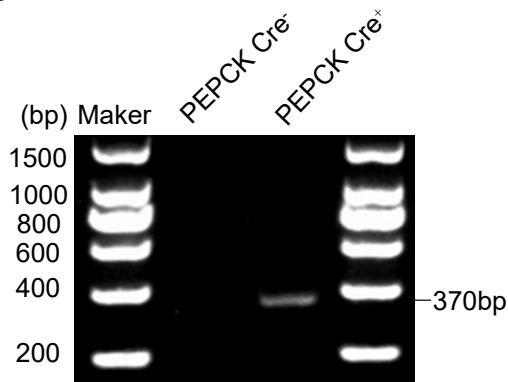

Supplement: Supplementary file 1 — Supplementary Material 1: Figure S1. Establishment and identification of PT-atg5 KO mice. (A) Breeding protocol for generating PT- atg5 KO mice. Male littermate mice, 8–10 weeks old, were used for experiments after genotypes were confirmed. (B) Representative images of Atg5 flox and PEPCK-Cre determination. [file 11658_2024_663_MOESM1_ESM.pdf]

A

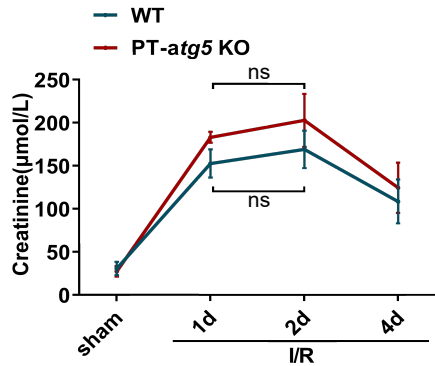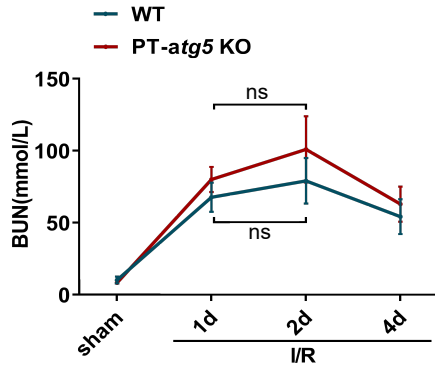

B

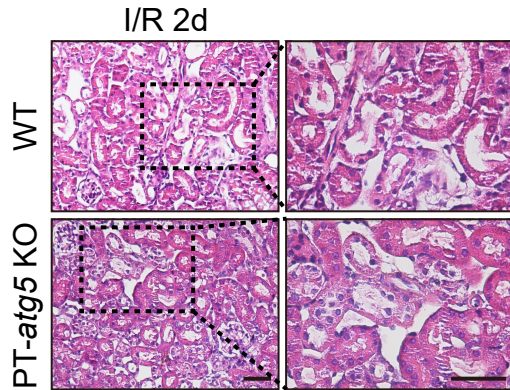

Supplement: Supplementary file 2 — Supplementary Material 2: Figure S2. The effect of tubular Atg5 deficiency on acute kidney injury after I/R. (A) Serum creatinine and BUN levels during acute injury phase (n = 5). (B) H&E staining images of the kidney at 2 days after I/R. Scale bar, 50 μm. The values are expressed as mean ± SD. [file 11658_2024_663_MOESM2_ESM.pdf]

A

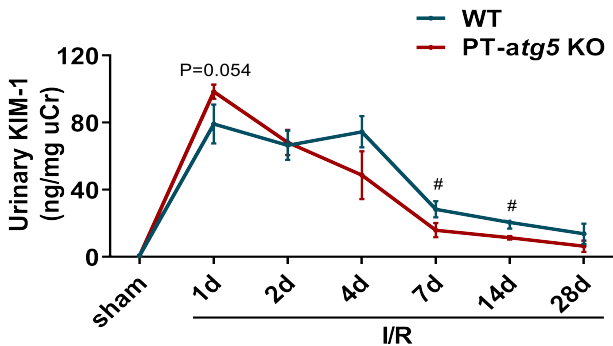

Supplement: Supplementary file 3 — Supplementary Material 3: Figure S3. Tubule Atg5 specific deletion affect the levels of urinary KIM-1 after I/R. Urine samples were collected at indicated times after sham surgery or after reperfusion following 25 min of bilateral ischemia. (A) Urinary KIM-1 normalized to urine creatinine (n = 3). The values are expressed as mean ± SD. # Represents a significant difference from the relevant wild-type group. # P <0.05. [file 11658_2024_663_MOESM3_ESM.pdf]

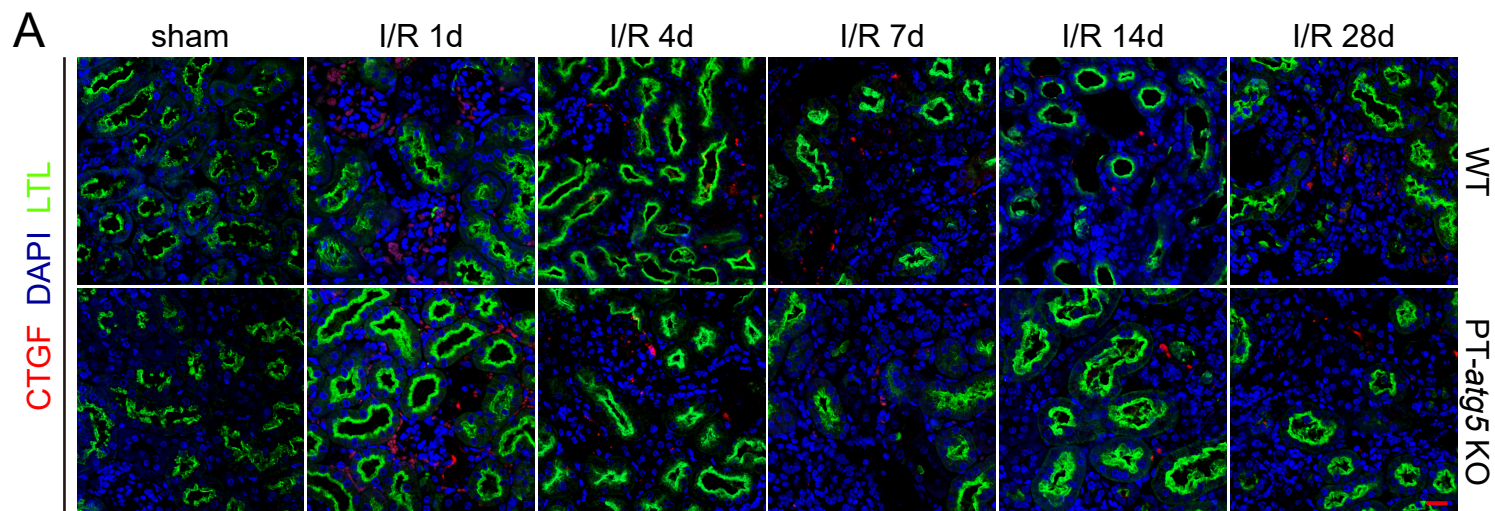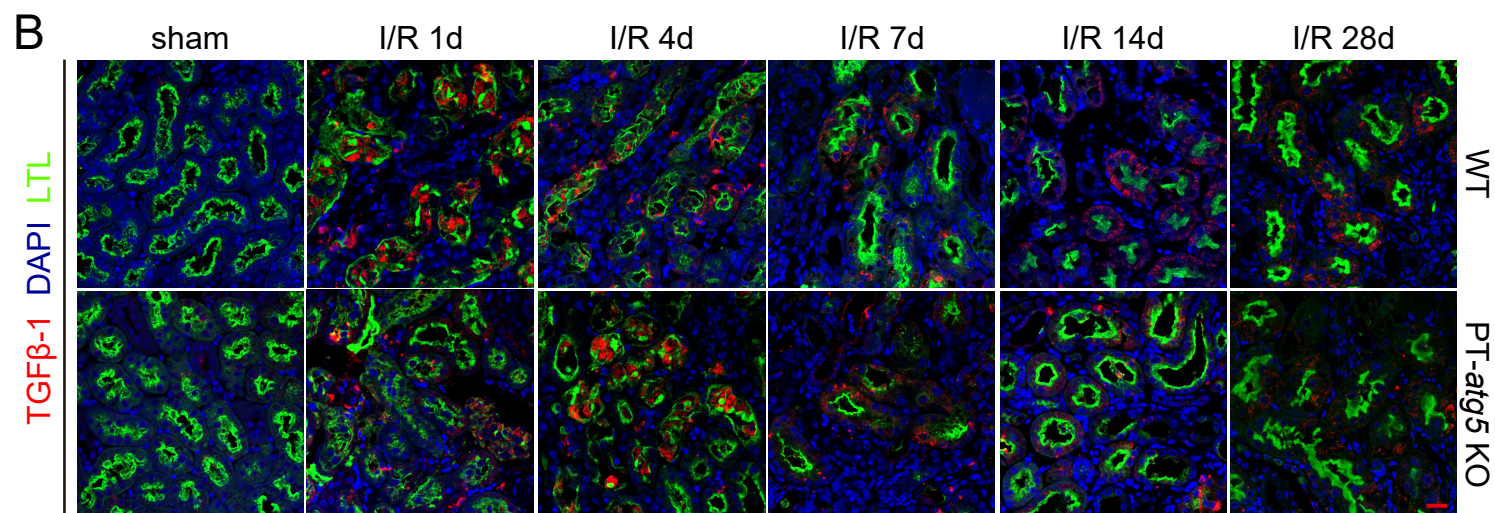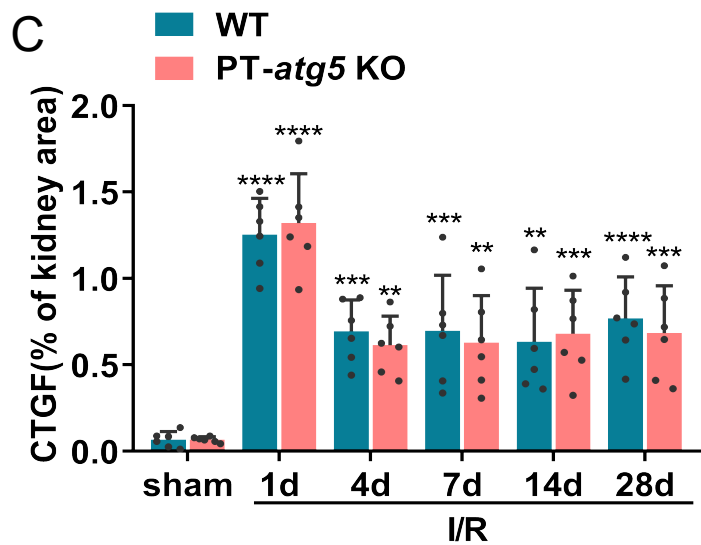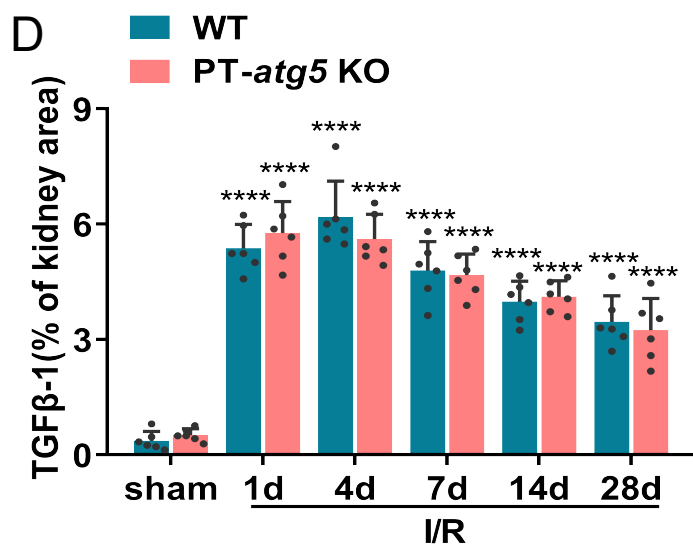

Supplement: Supplementary file 4 — Supplementary Material 4: Figure S4. Atg5 deficiency does not affect the production of CTGF and TGFβ1 in renal tubules during renal repair after I/R. Kidneys with or without bilateral renal I/R were collected at indicated time points and processed for immunofluorescence analysis of profibrotic factor CTGF and TGFβ1. (A) Immunofluorescence staining for CTGF (red). (B) Kidney slices were immunostained with antibody against TGFβ1 (red), stained using DAPI to visualize cell nuclei (blue), and LTL to visualize proximal tubules (green). Scale bars, 20 μm. (C) Quantification of CTGF-positive area (%). (D) Quantitative analysis of TGFβ1 positive area fraction. The values are expressed as mean ± SD (n = 6). * represents a significant difference from the sham group; # represents a significant difference from the relevant wild-type group. # or * P <0.05, ## or ** P <0.01, ### or *** P <0.001, #### or **** P <0.0001. [file 11658_2024_663_MOESM4_ESM.pdf]
