# Supplementary material for: Dynamic regulation of proximal tubular autophagy from injury to repair after ischemic kidney damage
Source: Cell Mol Biol Lett. 2024 Dec 5;29:151. doi: 10.1186/s11658-024-00663-w (PMC11619129; doi:10.1186/s11658-024-00663-w)
Supplement: Supplementary file 5 — Supplementary Material 5: Figure S5. Tubule Atg5 specific deletion does not affect the production of CTGF and TGFβ1 in renal tubules during UUO. Kidneys subjected to sham or UUO surgery were collected on day 14 to measure levels of profibrotic factors TGFβ1 and CTGF. (A) Representative immunofluorescence staining images with the CTGF antibody (red). (B) Representative TGFβ1 (red) immunofluorescence staining images of kidney slices. The cell nuclei were labeled with DAPI (blue). Proximal renal tubules were stained with LTL (green). Scale bars, 20 μm. (C) Quantification of CTGF-positive areas. (D) Quantitative analysis of TGFβ1 positive area fraction. Scale bars, 20 μm. The values are expressed as mean ± SD (n = 6). * represents a significant difference from the sham group; # represents a significant difference from the relevant wild-type group. # or * P <0.05, ## or ** P <0.01, ### or *** P <0.001, #### or **** P <0.0001. [file 11658_2024_663_MOESM5_ESM.pdf]

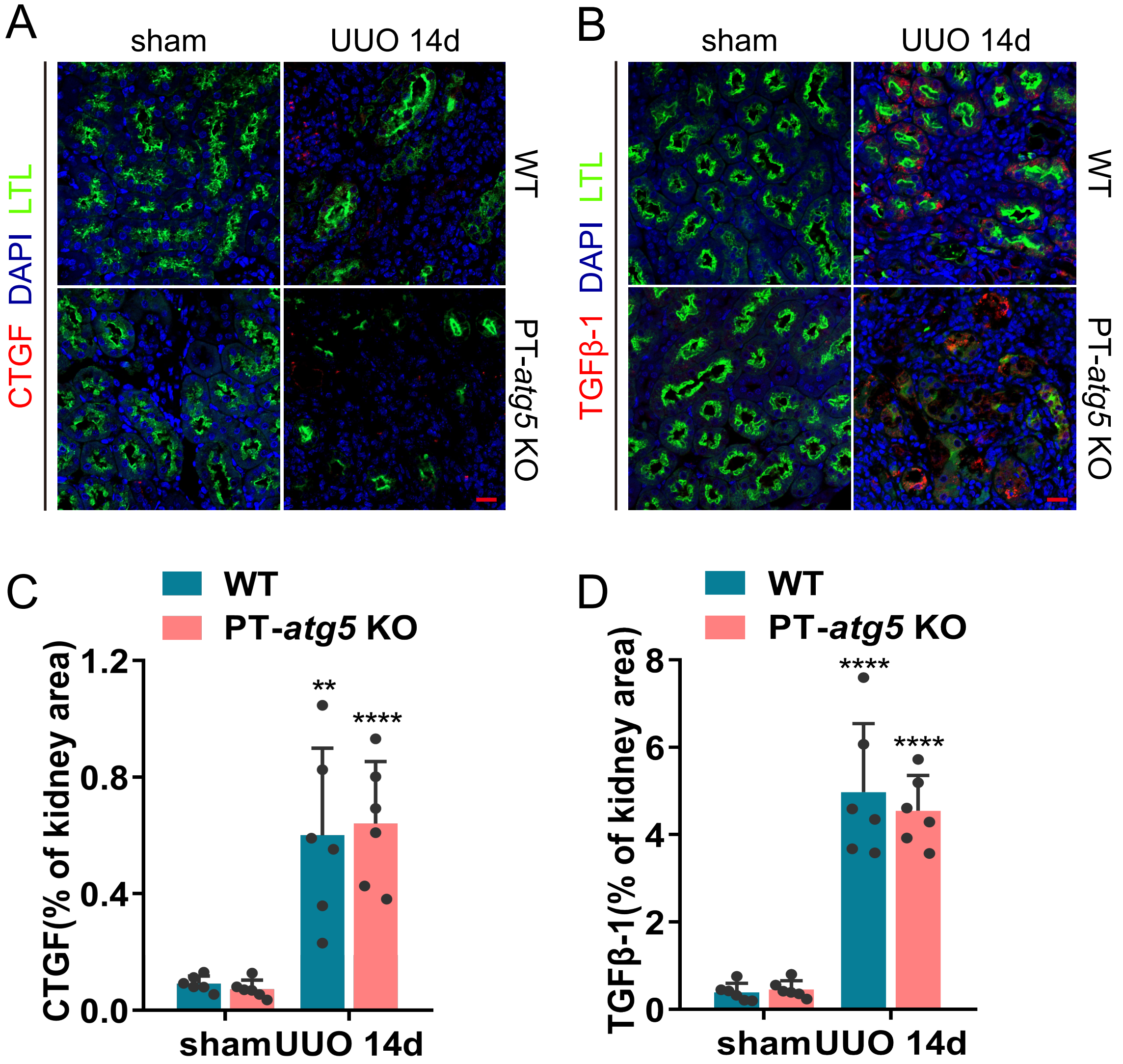
A
sham
UUO 14d
WT
PT-atg5 KO
CTGF DAPI LTL
B
sham
UUO 14d
WT
PT-atg5 KO
TGFβ-1 DAPI LTL
C
WT
PT-atg5 KO
CTGF(% of kidney area)
1.2
0.8
0.4
0.0
**
****
sham
UUO 14d
D
WT
PT-atg5 KO
TGFβ-1(% of kidney area)
8
6
4
2
0
****
****
sham
UUO 14d
